# The Correlation between Diffusion-Weighted Imaging and Histopathological Evaluation of 356 Prostate Biopsy Sites in Patients with Prostatic Diseases

**DOI:** 10.5402/2012/252846

**Published:** 2012-06-26

**Authors:** R. Kilinç, O. G. Doluoglu, B. Sakman, D. S. Ciliz, E. Yüksel, O. Adsan, M. Cetinkaya

**Affiliations:** ^1^Department of Urology, II Clinic, Ankara Numune Education and Research Hospital, Ankara, Turkey; ^2^Department of Radiology, I Clinic, Ankara Numune Education and Research Hospital, Ankara, Turkey; ^3^Department of Urology, I Clinic, Ankara Numune Education and Research Hospital, Ankara, Turkey

## Abstract

*Purpose*. The aim of this study is to investigate the reliability of diffusion MRI for detection of cancer foci by comparing diffusion-weighted imaging (DWI) results and pathology results of prostate biopsy sites. *Methods*. Of the patients who applied with lower urinary tract symptoms, 36 patients who had suspected DRE and/or PSA ≥2.5 ng/mL were included in the study. Patients underwent DWI prior to 10 cores-prostate biopsy. 356 biopsy cores were obtained from the patients. Foci from the patients with prostate cancer were labeled as malignant or benign foci, likewise foci from the patients with benign pathology were grouped as BPH and inflammation foci. Apparent diffusion coefficients (ADCs) of biopsy groups were compared with each other in order to measure the reliability of DWI in detection of PCa foci. *Results*. When ADC values of adenocarcinoma foci and BPH foci were compared, a statistically significant difference was found (*P* < 0.001). When ADC values obtained from adenocarcinoma foci and chronic inflammation foci are compared, the difference between two groups is statistically significant, too (*P* < 0.001). *Conclusions*. Biopsies focused on suspected regions after formation of ADC maps by means of DWI would provide to start definitive treatment immediately as well as being beneficial to prevent morbidity related to repeated prostate biopsies.

## 1. Introduction

Prostate carcinoma is the most common cancer type among men and is the second leading cause of cancer-related deaths following lung cancer [1].

 Today diagnosis is made by prostate needle biopsy accompanied by transrectal ultrasonography (TRUS) [2]. However, positive predictive value (PPV) of elevated serum PSA levels and transrectal ultrason guided biopsy are relatively low. Only 25% of the patients who have gray-zone PSA levels were detected to have prostate cancer [3]. PPVs of PSA and DRE were reported as 42% and 31%, respectively [4]. 

In patients with high serum PSA levels, to avoid from morbidity related to unnecessary biopsies without glossing over prostate cancer is a topic that should be thought about. Unnecessary biopsies might be avoided by correct detection and localization of prostate cancer; thus, novel techniques are being developed in order to detect prostate cancer and to evaluate its location [5, 6]. A method that is used in pathologies of different organs in recent years and subjected to research is diffusion-weighted imaging (DWI) [6–8].

The aim of this study is to investigate the reliability of diffusion MRI in detection of prostate cancer foci by comparing DWI results and pathology results of prostate biopsy sites in patients who are planned to undergo TRUS-guided prostate biopsy due to high PSA levels and abnormal DRE findings.

## 2. Methods

Diffusion-weighted MRI prior to TRUS-guided prostate biopsy was recommended and applied to the patients who were selected among the patients who applied to Ankara Numune Research and Training Hospital second Urology Clinic between November 2007 and February 2010 with lower urinary tract symptoms and found to be at risk of prostate cancer (PCa) with a PSA level ≥2.5 ng/mL and/or presence of a suspected nodule on digital rectal examination (DRE). As the result of this assesment, patients and prostate biopsy foci were divided according to their pathologic diagnosis and included in the study. Patients who underwent prostate biopsy previously, who had a history of open or endoscopic surgery targeted to prostate and urinary bladder and who received 5-alpha-reductase inhibitors for BPH were excluded. Patients who were not suitable for MRI were also excluded.

Thirty-six patients who accepted to participate in the study underwent MRI and afterwards TRUS-guided systematic 10-cores prostate biopsy was performed. Prostate biopsy was taken from 356 foci from 36 patients. Six quadrant biopsy could be taken from one patient as he could not tolerate the procedure. Thirty-six patients were evaluated as cancer and benign (BPH, inflammation) according to biopsy results. Prostate biopsy foci of patients who have had PCa were divided into two groups as malignant and benign foci. Additionally prostate biopsy foci of the patients with benign results were evaluated as BPH and inflammation.

Apparent diffusion coefficient (ADC) of all foci of patients whose pathologic diagnosis was prostate cancer was compared with ADCs of all foci of patients with benign pathologies in order to measure the reliability of diffusion MRI for detection of prostate cancer foci. Furthermore, ADCs of adenocancer foci of patients whose pathologic diagnosis was prostate cancer were compared with ADCs of benign foci of the same patients. ADCs of malignant foci of PCa patients and ADCs of BPH and inflammation foci of benign patients were compared. ADC measurements done as the result of DWI of prostate were performed by taking prostate locations determined as the result of prostate biopsies.

### 2.1. Magnetic Resonance Imaging

Prostate MRIs of the patients who accepted to take part in the study were conducted in Magnetic Resonance Unit of Ankara Numune Training and Research Hospital Department of Radiology. The study was conducted using 1.5T GE Signa Hispeed Excite MR system (General Electric, Milwaukee, WI). All cases were studied in supine position, head part so as to be close to magnet according to the used coil. Patients were prepared for the examination so as to their prostates were centered in 4-channel torso-PA coil. They were informed about the rules that they should obey in the course of the examination.

DWIs of prostate were done before TRUS-guided biopsy in order to eliminate the adverse effects of hemorrhage and inflammation on ADC values. No preparations were done like fasting and not to drink water prior to the study.

After taking 3-plane-localizer (pilot) imagings, diffusion-weighted imagings were taken from the prostates of the patients on axial plane. The patient was kept in touch by communicating with him prior to taking images. Images were began to be taken after these procedures. Examination took 30 seconds in total, 15 seconds for each prostate. b600 axial diffusion weighted echo-planer image (EPI) was taken from each patient. Parameters that were used in images are the following: Matrix: 128 × 128, NEX: 1.0, FOV: 30, cross-section thickness: 5 mm, intesectional space: 0, diffusion direction: all directions, TR: 8000, and TE: minimum

### 2.2. Image Analysis

After obtained diffusion-weighted images had been processed in working station of MR system, ADC maps were prepared for each patient. ADC values were measured as mm^2^/sn. ADC values were measured with 10 mm^2^ circular examination areas (region of interest (ROI)) from prostate biopsy sites. In other words, ADC values were determined by knowing biopsy data of the patients.

### 2.3. Statistical Analysis

SPSS ver. 13 (SPSS Ing., Chicago, IL, USA) package program was used for statistical analysis of the data. Mann-Whitney *U* test, Student's *t*-test, analysis of variance (ANOVA), and post-hoc analysis were used for analysis of measured data. *P* < 0.05 was taken statistically significant.

## 3. Results

Mean age ± standard deviation (SD) of the patients included in the study was found as 64.3 ± 7.2 (min–max: 53–86), mean ± SD of serum total PSA values was found as 8.05 ± 5.77 (min–max: 2.82–35.04 ng/mL), and mean ± SD of prostate volumes was found as 46.8 ± 19.4 (min–max: 21–90 gr).

Of the PCa patient group, mean age ± SD was found as 69 ± 8.3 (min–max: 58–86), mean ± SD of serum total PSA was found as 10.75 ± 9.25 (min–max: 3.62–35.04 ng/mL), and mean ± SD of prostate volumes was found as 44.7 ± 8.9 (min–max: 21–88 gr).

Of the patients in benign pathology group, mean age ± SD was found as 62.5 ± 6 (min–max: 53–77), mean ± SD of serum total PSA values was found as 7.02 ± 3.45 (min–max: 2.82–18.90 ng/mL), and mean ± SD of prostate volumes was found as 47.6 ± 19.9 (min–maks: 22–90 gr) (Table 1).

Of 36 patients, PCa was detected in 10 and benign pathologies (BPH, inflammation) were detected in 26. Of 100 foci obtained from 10 PCa patients, adenocancer was found in 25, and benign pathologies were found in 75. Biopsies were taken from a total of 256 foci of 26 patients who were detected to have benign pathologies according to prostate pathology results. Of them, BPH was detected in 212 and inflammation was detected in 44.

In measurements of ADC maps, mean ADC value obtained from prostates of patients with benign pathologies was detected as 1.65 × 10^−3^ ± 0.18 × 10^−3^ mm^2^/sn. Similarly, mean ADC value obtained from prostates of patients with PCa was detected as 1.51 × 10^−3^ ± 0.19 × 10^−3^ mm^2^/sn. When ADC values obtained from patient groups with benign pathologies and PCa are compared, the difference between two groups is statistically significant (*P* < 0.05) (Table 2).

In measurements done in ADC maps, mean ADC value of adenocarcinoma foci of patients that PCa was detected was found as 1.34 × 10^−3^ ± 0.43 × 10^−3^ mm^2^/sn and mean ADC value of benign foci of the same patients was found as 1.57 × 10^−3^ ± 0.29 × 10^−3^ mm^2^/sn. When ADC values obtained from adenocarcinoma and benign foci are compared, the difference is statistically significant (*P* < 0.05) (Table 3).

In measurements done in ADC maps prepared similarly, mean ADC value of adenocarcinoma foci of patients in whom PCa had been detected was found as 1.34 × 10^−3^ ± 0.43 × 10^−3^ mm^2^/sn, mean ADC value of BPH foci of patients with benign pathologies was found as 1.63 × 10^−3^ ± 0.28 × 10^−3^ mm^2^/sn   and mean ADC value of chronic inflammation foci of patients with benign pathologies was found as 1.76 × 10^−3^ ± 0.24 × 10^−3^ mm^2^/sn. When ADC values obtained from adenocarcinoma foci and BPH foci were compared, a statistically significant difference was found between two groups (*P* < 0.001). When ADC values obtained from adenocarcinoma foci and chronic inflammation foci are compared, the difference between two groups is statistically significant, too (*P* < 0.001). 

## 4. Discussion

PCa screening is done using DRE and PSA. While prostate cancer had been diagnosed in metastatic stages before 1987, when PSA measurements were not widely used, it is more commonly diagnosed in localized stages at present.

Positive predictive values of PSA and DRE were detected as 42% and 31%, respectively. This ratio increases to 60% when they are evaluated together [4]. The upper limit for serum PSA has traditionally been accepted as 4 ng/mL. However, biopsies have become widespread for values of ≥2.5 ng/mL. But probability of prostate cancer cannot be ruled out with only PSA values. Additionaly, high PSA values are not specific for prostate cancer.

In the study of Thompson et al. they diagnosed prostate cancer in 449 (15.2%) of 2950 patients whose total PSA (TPSA) values were below 4 ng/mL and found that 67 of them had high-grade cancers [9]. Prostate cancer was detected in a ratio of 6.6% even when TPSA was below 0.5 ng/mL, and, more importantly, 12.5% of them were found to be high-grade cancers. Thus it, is not possible to detect a 100% confident low limit for TPSA.

Most of the PCa are located in peripheral zone of the prostate and it may be diagnosed with DRE when its volume is 0.2 mL and above. A suspected DRE is the definite indication for prostate biopsy. In aproximately 18% of all patients, prostate cancer is detected with only suspected DRE independently from PSA levels [10].

Despite the two-fold probability of presence of cancer in hypoechoic regions in TRUS compared to isoechoic regions, 25%–50% of prostate cancers would be overlooked if biopsy is taken from only hypoechoic regions. Thus, TRUS is not recommended as a screening method for localized early stage prostate cancer. Main role of TRUS is to provide a correct site sampling of prostate tissue in a risky man for prostate cancer [11].

Serum total PSA levels and TRUS-guided prostate biopsy in the light of DRE findings that are accepted as the gold standard for diagnosis of prostate cancer have some shortcomings. In this technique, randomized biopsy method is used instead of targeted biopsy. Many disadvantages appear due to randomized method. These may include an increase in complication risk due to unnecessary sampling of prostate tissue, overlooking of cancer focus, and difficulty in determining previous biopsy sites in repeated biopsies of patients whose high PSA levels persisted despite negative biopsy results. Thus, an imaging modality is required in order to correctly diagnose prostate cancer and determine its location [12].

In recent years, a method that is used in pathologies of different organs and have been subjected to researches is DWI. DWI is sensitive to molecular transfer of water in biologic fluids due to randomized thermal movement of molecules. Microscopic movement includes molecular diffusion of water and microcirculation of blood in capillary net. MRI measures combined effect of both diffusion and capillary perfusion by means of apparent diffusion coefficient (ADC) [13].

The first use field of DWI is neuroradiology, it was especially used in pathologies like acute ischemic stroke, demyelinizing tumors, and intracranial tumors. DWI was used for characterization of abdominal organs and hepatic lesions, distinction of mailgnant and benign breast lesions, and evaluation of cystic ovarian masses by obtaining rapid imaging techniques. Clinical success of DWI caused its use area expanding towards prostate [13].

In this study, reliability of diffusion MRI in detection of prostate cancer foci by means of comparing DWI results and pathology results of prostate biopsy sites in patients who were planned to undergo TRUS-guided prostate biopsy beacuse of high PSA levels and/or abnormal DRE findings was investigated and significant results were obtained.

In the study of Sato et al. carried out with 29 patients with suspected prostate cancer, patients were divided into two groups as the ones that PCa was detected (*n*: 23) and not detected (*n*: 6) according to prostate biopsy results. DWI was performed prior to prostate biopsy. After biopsies, ADC maps were formed using DWIs according to histopathologic results of foci. As the result of these measurements, they found significantly lower ADC values in cancer including prostate tissues in both peripheral and transitional zones of each patient's prostate tissue [14]. Similarly in our study, when mean ADC values of adenocancer foci and benign foci were compared, a statistically significant difference was found (*P* = 0.015).

In the study of Issa carried out with 17 patients, mean ADC values were found statistically significantly lower in malignant peripheral zone tissues compared to the values of nonmalignant peripheral zone tissues [15]. Also in our study, mean ADC values of PCa foci and benign foci were compared and a statistically significant difference was found (*P* = 0.037).

In the study of Tamada et al. ADC values of 125 healthy volunteeer men obtained with DWI of peripheral and transitional zones and ADC values of 90 prostate cancer patients obtained with DWIs of cancerous peripheral zone and cancerous transitional zone were compared, and mean ADC values of patients who had cancer in both peripheral and transitional zones were found significantly lower compared to mean ADC values of peripheral and transitional zones of healthy volunteers [16]. In our study, mean ADC values of prostate cancer patients and patients with benign pathologies were compared and a statistically significant difference was found (*P* = 0.037).

Results of our study and the three studies mentioned above indicate that mean ADCs of foci that PCa had been detected on biopsy were statistically different from the ADC values of foci that PCa had not been detected. Normally, prostatic acini distributes radially into prostate from urethra, however, this order may be disrupted in cancerous tissue, malignant epithelial cells and glands may distribute irregularly, and internal structure of the malignant gland may be disrupted. Besides, water-rich aciner structures change over with cancer cells and cellular density and nucleus/cytoplasma ratio increases in cancerous tissues [15]. As the result, normal glandular structure of prostate disrupts in PCa and this glandular structure changes over with aggregated cancer cells and fibrotic stroma [17]. Because of the loss of glandular structure, interstitial space reduces and diffusion is limited. As DWI is sensitive to diffusion changes, this change appear with a decrease in ADC values [6]. We consider that the detection of lower ADC values in tissues with PCa compared to tissues with benign pathology may be explained with aforementioned changes in prostate morphology.

PCa is known to be located in transitional zone with a ratio of 21–24%. Especially transitional zone is targeted in biopsies of patients who have multiple negative biopsy results and high PSA levels. Small tumors (<2 cm^3^) may not be diagnosed even when transitional zone is targeted [14]. Because tumor volume is <0.5 cm^3^ in 55% of transitional zone cancers, diagnosis of transitional zone cancers may be made more correctly by imaging transitional zone with DWI and determining low ADC sites and directing biopsy towards those sites.

A repeated biopsy may be required for patients who had suspected findings in terms of PCa and whose first transrectal prostate biopsy result was negative and in whom suspicion of cancer continued. However, ratios of cancer detection reduces significantly after the second biopsy. In this patient groups, generating ADC maps with DWI and biopsies' focusing on suspected sites in terms of prostate cancer with low ADC values would both help protecting the patients from morbidities due to repeated biopsies and to begin definite treatment immediately by providing diagnosis without delay. Being expensive and time-consuming are the disadvantages of ADC despite these benefits.

## 5. Conclusion

 Ten or more cores prostate biopsy with TRUS guidance are the standard diagnostic method for PCa. However, a particular number of cancer cases could not be diagnosed on the first biopsy. According to our findings, DWI may increase the efficacy of prostate biopsy by distinguishing localization of cancer foci from the benign ones, minimizing the need for third or more biopsies and the related morbidity.

## Figures and Tables

**Table 1:** Ages, prostate volumes, and serum PSA levels of the patients and statistical relationships.

	Total	Benign	Malignant	*P* value
Number	36	26	10	
Age median (min–max)	63 (53–86) years	63 (53–77) years	69 (58–86) years	*P* = 0.031*
Total PSA median (min–max)	6.7 (2.8–35.0) ng/mL	6.7 (2.8–18.9) ng/mL	6.9 (3.6–35.0) ng/mL	*P* = 0.303
Volume median (min–max)	45 (21–90) gm	45 (22–90) gm	42 (21–88) gm	*P* = 0.664

TPSA: total prostate specific antigen, ^∗^statistically significant.

**Table 2:** Mean ADCs of groups study and statistical relationships.

	Prostate Ca (*n1*: 10 patients and 100 foci)	Benign pathology (*n1*: 26 patients and 256 foci)	*P* value
Mean ADC (×10^−3^ mm^2^/sec)	1.51 ± 0.19	1.65 ± 0.18	*P* = 0.037*

*n1*: patient number, ^∗^statistically significant.

**Table 3:** Mean ADCs of all foci and statistical relationships.

	AdenoCa Foci (*n2*: 25)	Prostate Ca Benign Foci (*n2*: 75)	BPH Foci (*n2*: 212)	Chronic Inflammation Foci (*n2*: 44)	*P* value
Mean ADC (×10^−3^ mm^2^/sec)	1.34 ± 0.43	1.57 ± 0.29			*P* = 0.015*
	1.34 ± 0.43		1.63 ± 0.28	1.76 ± 0.24
	1.34 ± 0.43		1.63 ± 0.28		*P* = 0.025*
	1.34 ± 0.43			1.76 ± 0.24	*P* = 0.0001*

*n2*: foci number, ^∗^statistically significant.
